# Integrin *α*6 Indicates a Poor Prognosis of Craniopharyngioma through Bioinformatic Analysis and Experimental Validation

**DOI:** 10.1155/2022/6891655

**Published:** 2022-10-11

**Authors:** Yanfei Jia, Wentao Wu, Youchao Xiao, Kefan Cai, Songbai Gui, Qiang Li, Tian Li

**Affiliations:** ^1^Department of Neurosurgery, Beijing Tiantan Hospital, Capital Medical University, Beijing 100070, China; ^2^Department of Neurosurgery, The Second Affiliated Hospital of Lanzhou University, Lanzhou 730000, China; ^3^School of Basic Medicine, Fourth Military Medical University, Xi'an 710032, China

## Abstract

**Background:**

Craniopharyngioma (CP) is a benign slow-growing tumor. It tends to affect children, and the number of patients is on rise. Considering the high morbidity and mortality of CP, it is urgent and pivotal to identify new biomarkers to uncover the etiology and pathogenesis of CP.

**Methods:**

The “limma” package was utilized to calculate the data from the Gene Expression Omnibus (GEO) database. Based on differentially expressed genes (DEGs), gene ontology and pathway analysis were deduced from the DAVID web tool. Further, we constructed a protein-protein interaction (PPI) network. Weighted correlation network analysis (WGCNA) was utilized to build a coexpression network. Finally, Western blotting and survival analysis were performed to examine the expression level of important metabolism-related genes.

**Results:**

Three hundred and eighty-four DEGs were identified between normal tissues and CPs from the GSE94349 and GSE26966 datasets. The Venn diagram for DEGs and hub genes in the ‘turquoise' module revealed four key genes. Finally, the outcome of the survival analysis suggested that Integrin *α*6 (ITGA6) significantly affected the overall survival time of the patients with CP.

**Conclusion:**

IGTA6, as a metabolism-related molecule, was found to be substantially related to the overall survival of patients with CP.

## 1. Introduction

Craniopharyngioma (CP) is a locally aggressive tumor with a low histological grade (WHO I grade) [1], mostly occurs in the sellar and suprasellar regions [2]. Globally, the incidence rate of CP in children's ranged the third of intracranial tumors and it is also the most common nonneuroepithelial neoplasm in the hypothalamus and pituitary regions [3]. Despite the benign histologic appearance [4], due to invasion to important structures around the tumor, such as the optic chiasma, Willis ring, pituitary, and hypothalamus [5], hence, symptoms and signs of hypothalamic and pituitary dysfunctions are evident in patients with CP. Currently, surgery is the most effective treatment; however, the complexity of CP creates challenges for surgery treatment and leads to a high mortality rate. Difficulties still exist in the removal of the tumor owing to the anatomical structure adjacent to the suprasellar region and adhesion around tumor with the surrounding tissues. Some controversies regarding the etiology, histology, and pathology of CP, as also the optimal treatment strategy persist [3]. In short, it is urgent and important to identify new biomarkers to uncover the etiology and pathogenesis of CP.

Microarray-based high-throughput platform is a promising and efficient technique, which is widely used to screen epigenetic or genetic alternations and identify cancer biomarkers [6, 7]. Comprehensive calculation of data provides key avenues to explore the mechanisms of tumors [8]. Several gene expression profiling microarrays have been employed to identify the differentially expressed genes (DEGs) in CP, and some bioinformatic methods have been used to analyze the data. In this study, we processed raw data of CP samples in the GSE94349 and GSE26966 datasets downloaded from GEO. DEGs were analyzed, and WGCNA was performed to elucidate the possible mechanisms underlying CP more clearly.

## 2. Methods

### 2.1. Microarray Data

Two gene expression profiling datasets (GSE94349 and GSE26966 from GPL55999 platform), acquired from the GEO, comprise 9 normal pituitary tissues and 9 CP tissues. The two datasets were chosen for integrated analysis owing to their same platform.

### 2.2. Data Processing

We used the ‘limma' package (V3.29.0) with standard data processing conditions to identify DEGs. The cut-off criteria were *p* < 0.05 and |logFC| ≥ 2. Subsequently, the DEGs were analyzed by Gene Ontology (GO) term enrichment analysis and Kyoto Encyclopedia of Genes and Genomes (KEGG) pathway analysis using the ‘clusterprofiler' package (V4.4.3) in R V3.5.5. The protein-protein interaction (PPI) network was constructed using STRING Database (http://string-db.org/) and the Cytoscape software. Subsequently, the WGCNA package (V1.61) was employed to search the correlations among genes and identify the significantly correlated gene modules. The soft thresholding power was set at 6.

### 2.3. GO Term Annotation and KEGG Pathway Enrichment Analyses

We utilized clusterProfiler to perform GO and KEGG analyses, for a detailed comprehension of the DEGs. *p* < 0.05 was considered a significant enrichment.

### 2.4. Protein-Protein Interaction (PPI) Network Construction

The DEGs used for constructing PPI network were obtained through the ‘limma' package. The Cytoscape software (V3.8.1) was thereafter employed to analyze the interactive relationships among the candidate proteins [9]. The “Molecular Complex Detection” (MCODE) module (V2.0.2) was used to detect densely connected regions in large PPI networks that may represent molecular complexes [10].

### 2.5. Weighted Correlation Network Analysis (WGCNA)

Weighted correlation network analysis (WGCNA) was used for identifying modules of highly correlated genes, summarizing clusters using the module eigengenes or an intramodular hub gene, and correlating modules to one another and to the external sample traits (using eigengene network methodology) [11]. We used the WGCNA package to construct the gene coexpression network, identify modules, and finally obtain the genes in the modules of interest.

### 2.6. Identification of Key Genes

The final key genes were identified as the intersecting genes between those in the ‘turquoise' module from WGCNA and DEGs.

### 2.7. Tissue Collection

A total of 21 human CP tissues were acquired from the patients, and 10 normal pituitary tissues from other patients with common pediatric brain tumor types. No local or systematic neoadjuvant radiotherapy, or/and chemotherapy, and targeted therapy were managed. The study design was approved by the Research Ethics Committee of Lanzhou University (Lanzhou, Gansu, PR China) and all patients enrolled in this study provided signed informed consent.

### 2.8. Western Blotting

Initially, we selected four of the 21 human CP tissues and four normal pituitary tissues, as previously described. Total proteins of each sample were extracted with Cell lysis buffer for Western and IP (Beyotime Biotechnology, China), followed by quantification using bicinchoninic acid (BCA) kit (Beyotime Biotechnology, China). After being separated by 10% sodium dodecyl sulfate polyacrylamide gel electrophoresis (SDS-PAGE), the total protein was transferred onto the polyvinylidene fluoride (PVDF) membranes (Beyotime Biotechnology, China) which were then blocked for 1 h. Thereafter, the membranes were incubated first with primary antibodies against at 4°C overnight and then with a horseradish peroxidase-conjugated secondary antibody for 2 h. Later, protein-antibody complexes were visualized and analyzed using ECL chemiluminescent solution (Beyotime Biotechnology, China). Finally, the ImageJ software (Rawak Software, Germany) and GraphPad Prism (version 7, GraphPad Software, San Diego, USA) were used to analyze the grayscale values of the bands.

### 2.9. Survival Analysis

The survival analysis was performed of all 21 CP samples using the SPSS version 22.0 software (IBM Corp. Chicago, IL, USA). Kaplan-Meier curve and Cox's proportional hazards regression model were performed to analyze the overall survival, and the differences were analyzed for significance using the log-rank test. The statistically significant modules were defined as those with *p* < 0.05.

## 3. Results

### 3.1. Identification of Aberrant DEGs in CP

**Figure 1:**
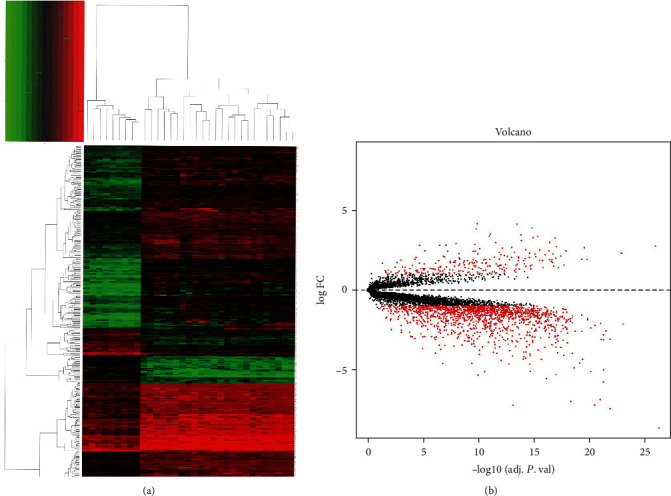
Heatmap and volcano plot of DEGs. (a) Heatmap of the differently expressed genes according to the values of |logFC| > 2. (b) Volcano map of differently expressed genes between CP tissues and normal pituitary tissues.

We used the ‘limma' package with the preprocessing parameters to analyze and obtain the DEGs among the GSE94349 and GSE26966 datasets. Using *p* < 0.05 and |logFC| ≥ 2 as the threshold criteria, a total of 384 DEGs were identified, including 56 downregulated and 328 upregulated genes in CP tissues as compared to the normal pituitary tissues (Figure 1(a)). These DEGs are shown in the volcano map (Figure 1(b)).

### 3.2. GO Functional Enrichment Analysis

Significant terms from GO enrichment analysis using DAVID are listed in Table 1. All the significant genes with a low expression are listed in the table. These genes were enriched in the biological processes (BP) involved in negative regulation of cilium assembly, endothelial cell apoptosis, regulation of transcription, DNA template, calcium ion transmembrane transport, and eye photoreceptor cell development. The results from DAVID analyses showed that there were no genes with a low expression enriched in molecular functions (MF) and cell components (CC). For the genes with a high expression, Table 1 shows the corresponding top five significant GO enrichment terms analyzed in DAVID. Molecular functions were enriched in cell adhesion molecule binding, glycoprotein binding, signal transducer activity, cadherin binding involved in cell-cell adhesion, and protein binding. Additionally, cellular component enrichment was found in focal adhesion, extracellular exosome, cell surface, extracellular space, and extracellular matrix while biological processes enrichment was found in cell adhesion, extracellular matrix organization, single organismal cell-cell adhesion, cellular response to low-density lipoprotein particle stimulus, and wound healing.

### 3.3. KEGG Pathway Analysis

As shown in Table 2, the results of KEGG suggested genes with a low expression were significantly enriched in pathways including adrenergic signaling and pancreatic secretion in cardiomyocytes. Genes with a high expression demonstrated enrichment in pathways of cancer, focal adhesion, proteoglycans in cancer, leishmaniasis, lysosome, PI3K-Akt signaling, and arrhythmogenic right ventricular cardiomyopathy (ARVC).

### 3.4. PPI Network and Cluster Analysis

**Figure 2:**
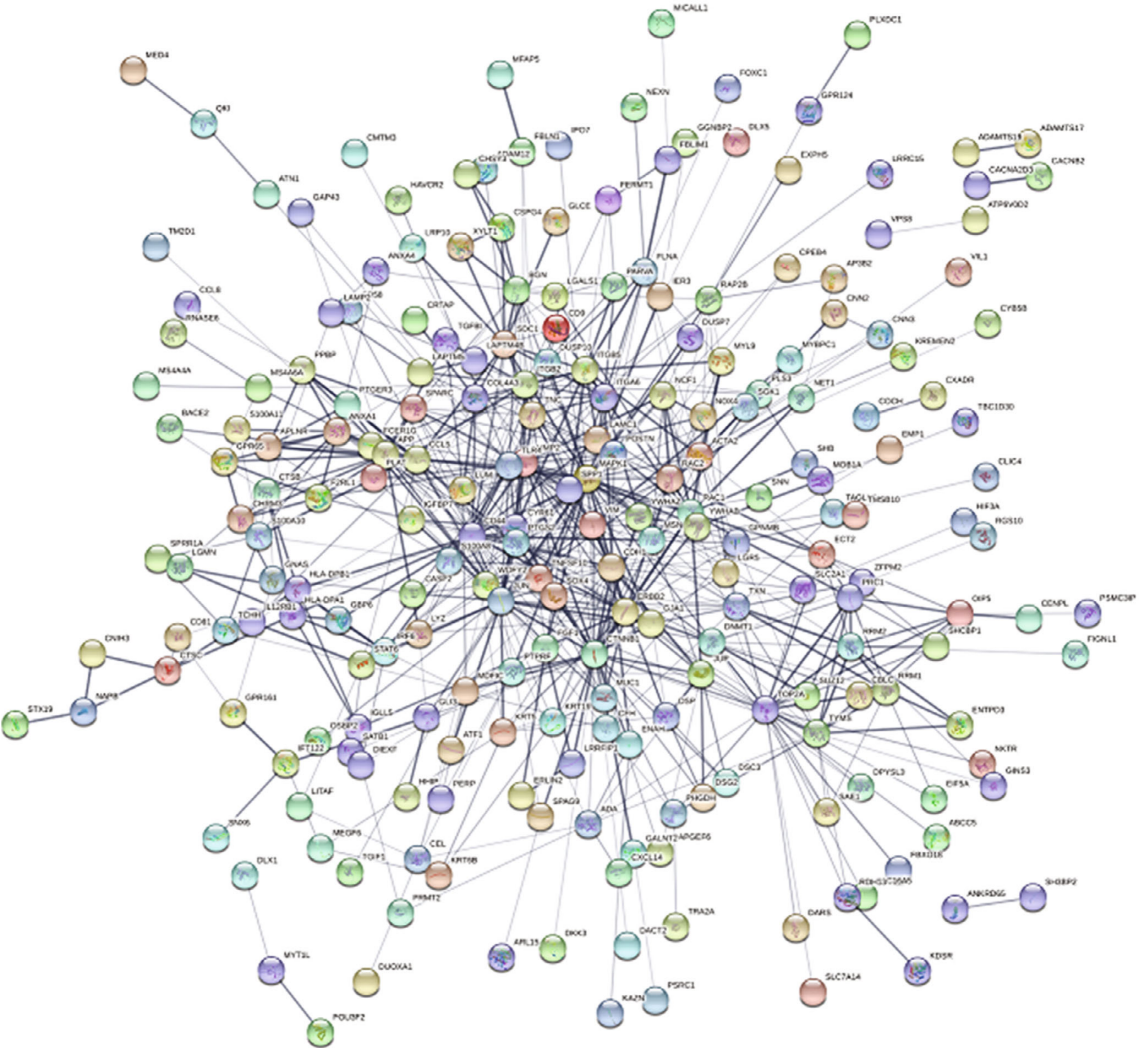
Cluster analysis of the PPI network. Three-hundred and differently eighty-four expressed genes were filtered into the DEGs' PPI network complex.

The PPI network was constructed using the STRING database. The Cytoscape software was used to analyze the interactive relationships among the candidate proteins. Module analysis was conducted using MCODE (Table 3). In the clusters, the following 29 genes: CCL5/PPBP/APLNR/ANXA1/GPR65/F2RL1/PTGER3/CHRM3/APP/GBP6/HLA-DPA1/HLA-DPB1/CD44/IRF6/TOP2A/ENTPD3/RRM1/TYMS/RRM2/XYLT1/BGN/SDC1/CSPG4/LRRFIP1/FGF1/ERLIN2/ITGA6/YWHAB/YWHAZ, were found to form the hub according to the MCODE findings (Figure 2).

### 3.5. WGCNA

**Figure 3:**
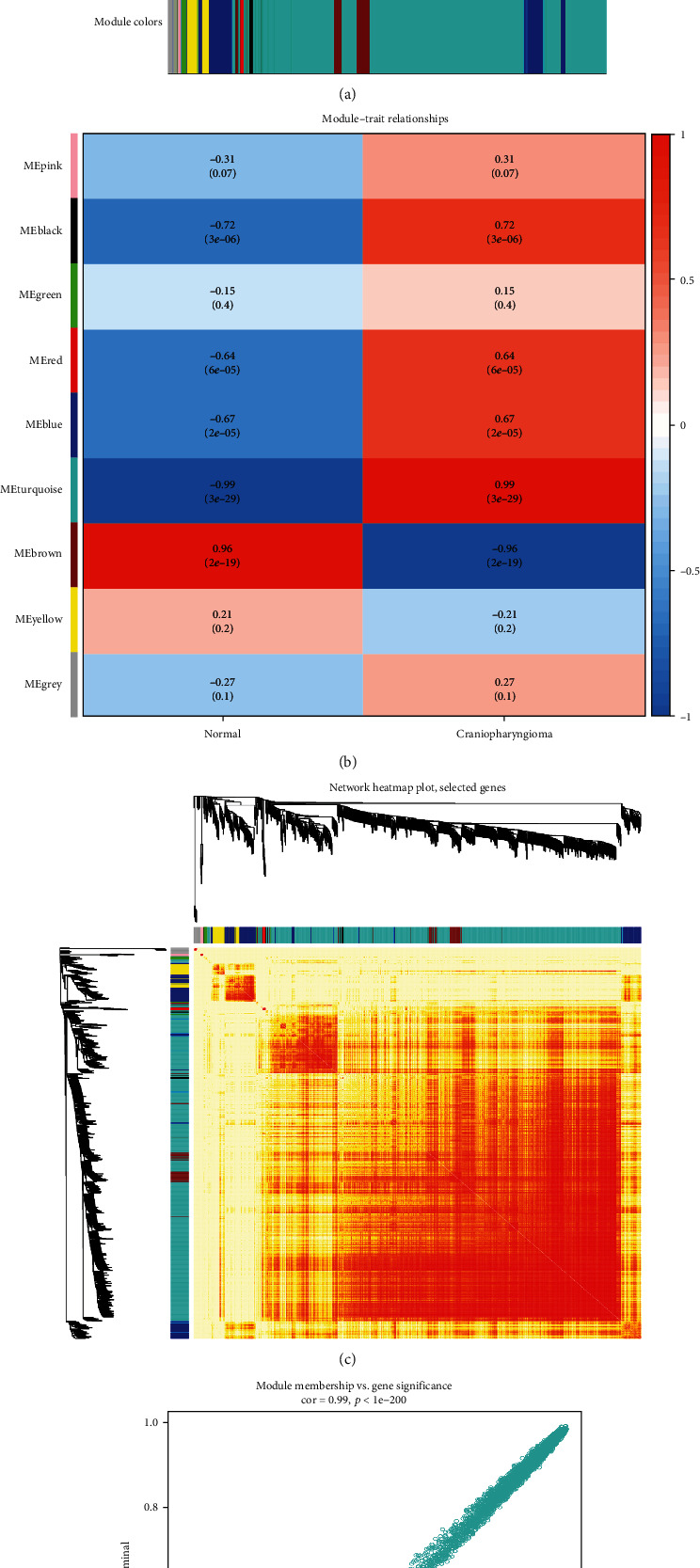
WGCNA for the GSE94349 and GSE26966 datasets. (a) The top image shows a gene dendrogram, and the bottom image shows the gene modules with different colors. (b) Correlation between modules and traits. The upper number in each cell refers to the correlation coefficient of each module in the trait, and the lower number is the corresponding *p* value. Among them, the turquoise modules were the most relevant modules with cancer traits. (c) A heatmap of 1,000 genes was selected at random. The intensity of the red color indicates the strength of the correlation between pairs of modules on a linear scale. (d) A scatter plot of CP tissues and normal pituitary tissues in the turquoise module. Intramodular analysis of the genes found in the turquoise module, which contains genes that have a high correlation with cervical cancer, with *p* < 1*e* − 200 and correlation = 0.99.

Among the modules, the ‘turquoise' one was found to be the most relevant for the cancer traits (Figures 3(a) and 3(b)). A total of 1,000 genes were selected at random for plotting the heatmap (Figure 3(c)). As shown in Figure 3(d), the ‘turquoise' module showed a high correlation. The genes in this module were then selected as the hub genes with a cut-off of correlation ≥ 0.5. Finally, 205 hub genes were identified from the chosen ‘turquoise' module.

### 3.6. Key Genes Identified among Hub Genes in Turquoise Module and DEGs

**Figure 4:**
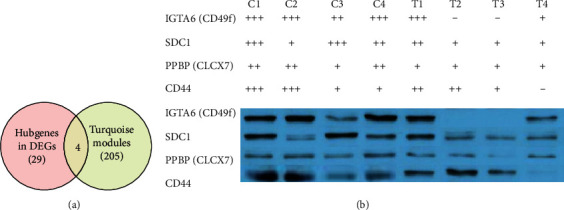
The key intersecting genes obtained from Venn diagram of DEGs and verified by Western blot. (a) A Venn diagram of DEGs and hub genes in the turquoise module shows 4 key intersecting genes. (b) Results verified by Western blot and obtained from the selected 8 samples. Abbreviations: C1-C4: craniopharyngioma tissues No.1-No.4; P1-P4: pituitary tissues No.1-No.4.

To obtain valuable clues from these data, key genes were identified from among the hub genes in the turquoise module and DEGs. In total, four key genes were obtained, namely PPBP, CD44, SDC1, and ITGA6 (Figure 4(a)).

### 3.7. Western Blotting and the Survival Curve Analyses

**Figure 5:**
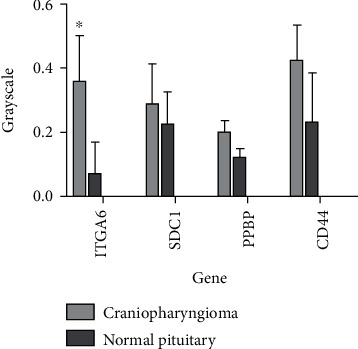
Grayscale value analysis for Western blotting. PPBP (CXCL7), CD44, SDC1, and ITGA6 (CD49f) protein levels are shown for the craniopharyngioma tissues and pituitary tissues. ^∗^*p* value < 0.05.

From the selected 8 tissues, only ITGA6 showed a low expression in all normal pituitary tissues (Figure 4(b)). Besides, in the tissues C1, C2, and C4, ITGA6 was overexpressed. As shown in Figure 5, the expression of ITGA6 was significantly different between the CP and pituitary tissues.

**Figure 6:**
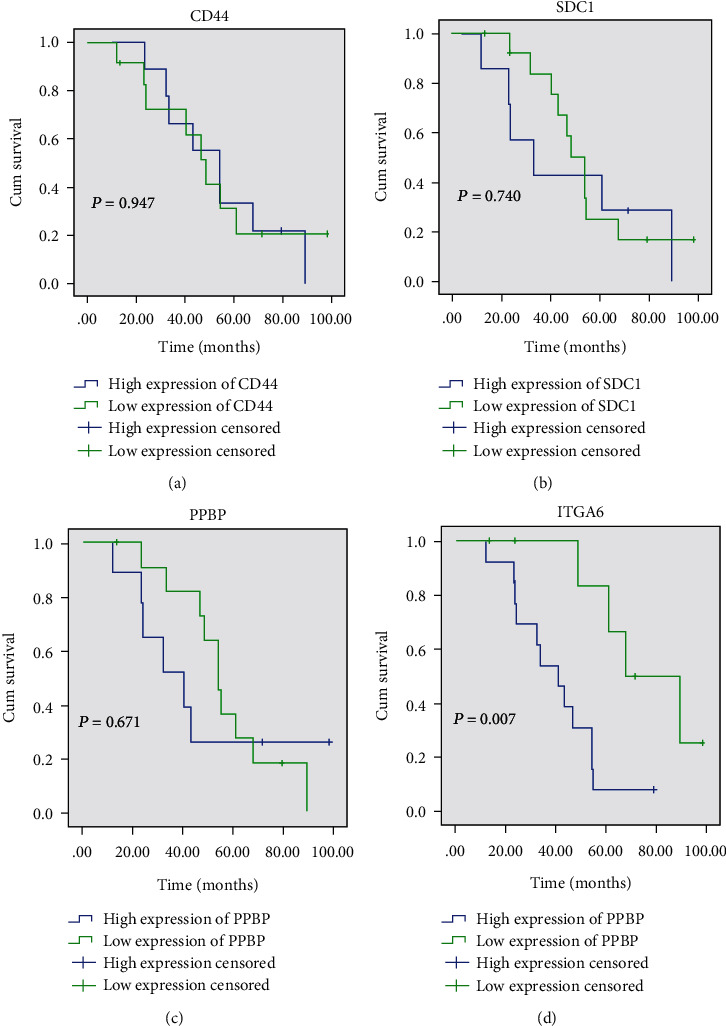
Survival analysis according to the levels of CD44, SDC1, PPBP, and ITGA6 expressions. (a) Effects of CD44 expression on craniopharyngioma patient survival. (b) Effects of SDC1 expression on craniopharyngioma patient survival. (c) Effects of PPBP expression on craniopharyngioma patient survival. (d) Effects of ITGA6 expression on craniopharyngioma patient survival.

Further, survival analysis for these four genes was employed to evaluate their effects on the overall survival of patients with CP. No significant differences were obtained in patients with CP showing differential levels of CD44, SDC1, and PPBP expressions (Figures 6(a)–6(c)). The results of the survival analysis indicated that the overall survival time of CP pantients could have significant differences between the high ITGA6 expression group and low ITGA6 expression group (Figure 6(d)).

## 4. Discussion

### 4.1. Main Finding

Neoplasm remains the main leading cause of death worldwide. Although CP is a benign slow-growing tumor [12], it appears partially aggressive and has an arachnoid interface with surrounding structures, thus rendering it incurable [12–15]. Hence, studies on invasion and the migration of CP are becoming increasingly popular. Previous studies show that the tumor microenvironment of craniopharyngioma has some particular characteristics, such as infiltration of leukocytes, a local abundance of adenosine triphosphate (ATP) and elevated levels of proinflammatory cytokines that are thought to be responsible for the local invasion. Yin et al. show that CXCL12/CXCR4 promotes proliferation, migration, and invasion of adamantinomatous CP via the PI3K/AKT signaling pathway [16]. In this study, using the GEO datasets and clinical samples, we aimed to identify new prognosis predictors for this disease.

### 4.2. Interpretation

Herein, we collected two GEO datasets and performed an integrated analysis using both DEGs and WGCNA to obtain valuable clues. A total of 384 DEGs were identified, including 56 downregulated genes and 328 upregulated genes in CP tissues compared to normal tissues. Enrichment analyses using GO annotation and KEGG pathways were subsequently performed to further analyze the functions of these genes. As suggested by the results of the DAVID analysis, genes with a high expression in CP tissues were enriched in biological processes of pathways in cell adhesion, extracellular matrix organization, and wound healing. Molecular functions from GO analysis showed enrichment in cell adhesion molecule binding, glycoprotein binding, and protein binding. This was reasonable as frequent cellular proliferation and loss of cell adhesion are hallmarks of malignant diseases including CP [17]. The results of the KEGG pathway enrichment analysis suggested significant enrichment in pathways including PI3K-Akt signaling and regulation of actin cytoskeleton. This was consistent with the fact that PI3K-Akt signaling is known to be frequently dysregulated in CP [16].

Four key genes identified from among the hub genes, PPBP (CXCL7), CD44, SDC1, and ITGA6 (CD49f), were selected for further experimental analyses. A total of four CP and four normal pituitary samples were processed for Western blotting, and we found the differential expressions of the four tested genes. After analyzing the results of Western blotting, ITGA6 was selected as the target gene to perform survival analysis.

Many studies show that ITGA6 is overexpressed in several carcinomas, including breast cancer, colorectal cancer, kidney cancer, and gallbladder carcinoma [18–20]. Moreover, the overexpression of ITGA6 suggests a poor prognosis in breast, colorectal, kidney, and gallbladder cancers. For instance, Zhang et al. report that ITGA6 overexpression is associated with invasion, metastases, and poor prognoses in human gallbladder carcinoma [19]. Several studies suggest that ITGA6 expression is associated with the progression and invasion of malignant lesions [21, 22].

Several researchers have invented new ways to inhibit the growth of cancers by targeting ITGA6 [23–25]. Wang et al. have successfully shown that miR-127-3p inhibits cellular growth and invasiveness by targeting ITGA6 in human osteosarcoma [24]. Laudato et al. report that P53-induced miR-30e-5p inhibits colorectal cancer invasion and metastases by targeting ITGA6 and ITGB1 [25]. However, the importance of ITGA6 in the CP remains unknown. Our findings demonstrated that the overexpression of ITGA6 was associated with the overall survival of patients with CP. The present study is, to the best of our knowledge, the first to investigate the association among the important biomarkers and characteristics of CP. Herein, the survival analysis indicated that ITGA6 was a poor prognostic factor for CP, which means patients with a high expression of ITGA6 had a significantly shorter overall survival relative to those with a low expression (*p* = 0.007).

### 4.3. Limitations

Although we found that IGTA6 affected the overall survival of patients with CP, some limitations to this investigation should be acknowledged. Further molecular experiments are needed to confirm the findings on the importance of ITGA6 for cellular invasion and proliferation in CP. Moreover, the regulatory factors for the expression of ITGA6 and the underlying pathways need further elucidation. For instance, Zhang et al. show that Twist2 promotes proliferation and invasion of kidney cancer cells by regulating ITGA6 and CD44 expressions in the ECM-receptor-interaction pathway [26]. We plan to address these in the future.

### 4.4. Conclusion

Several studies have been conducted over the past few years to investigate the pathogenesis of CP, yet little is known about the formation and progression of this disease [27–32]. In summary, our findings indicated that ITGA6 was significantly correlated with the survival of the patients. Moreover, it can serve as a clinical prognostic marker for CP. Further experiments shall be performed in the future to confirm our findings.

## Figures and Tables

**Table 1:** Gene ontology analysis for aberrant differentially expressed genes in craniopharyngioma.

Category	Term	Count	%	*p* value
Low expression				
GOTERM_BP_DIRECT	GO:1902018 ~ negative regulation of cilium assembly	2	3.64	0.01697
GOTERM_BP_DIRECT	GO:0072577 ~ endothelial cell apoptotic process	2	3.64	0.01697
GOTERM_BP_DIRECT	GO:0006355 ~ regulation of transcription, DNA-templated	9	16.36	0.02695
GOTERM_BP_DIRECT	GO:0070588 ~ calcium ion transmembrane transport	3	5.45	0.034121
GOTERM_BP_DIRECT	GO:0042462 ~ eye photoreceptor cell development	2	3.64	0.043071
High expression				
GOTERM_CC_DIRECT	GO:0005925 ~ focal adhesion	30	9.35	5.60*E* − 12
GOTERM_CC_DIRECT	GO:0070062 ~ extracellular exosome	90	28.12	2.77*E* − 11
GOTERM_CC_DIRECT	GO:0009986 ~ cell surface	28	8.75	1.64*E* − 07
GOTERM_CC_DIRECT	GO:0005615 ~ extracellular space	46	14.35	1.51*E* − 06
GOTERM_CC_DIRECT	GO:0031012 ~ extracellular matrix	18	5.63	5.44*E* − 06
GOTERM_BP_DIRECT	GO:0007155 ~ cell adhesion	28	8.75	1.07*E* − 08
GOTERM_BP_DIRECT	GO:0030198 ~ extracellular matrix organization	17	5.31	1.58*E* − 07
GOTERM_BP_DIRECT	GO:0016337 ~ single organismal cell-cell adhesion	9	2.81	2.60*E* − 04
GOTERM_BP_DIRECT	GO:0071404 ~ cellular response to low-density lipoprotein particle stimulus	4	1.25	3.43*E* − 04
GOTERM_BP_DIRECT	GO:0042060 ~ wound healing	8	2.50	3.43*E* − 04
GOTERM_MF_DIRECT	GO:0050839 ~ cell adhesion molecule binding	8	2.50	5.23*E* − 05
GOTERM_MF_DIRECT	GO:0001948 ~ glycoprotein binding	8	2.50	7.12*E* − 05
GOTERM_MF_DIRECT	GO:0004871 ~ signal transducer activity	13	4.06	9.79*E* − 05
GOTERM_MF_DIRECT	GO:0098641 ~ cadherin binding involved in cell-cell adhesion	15	4.69	2.15*E* − 04
GOTERM_MF_DIRECT	GO:0005515 ~ protein binding	166	51.88	5.92*E* − 04

**Table 2:** Results of KEGG enrichment for the differentially expressed genes.

Category	Term	Count	%	*p* value	Genes
Low expression					
KEGG_PATHWAY	hsa04972: pancreatic secretion	3	5.454545	0.009187	CEL, ATP2B3, GNAS
KEGG_PATHWAY	hsa04261: adrenergic signaling in cardiomyocytes	3	5.454545	0.019518	ATP2B3, CACNB2, GNAS
High expression					
KEGG_PATHWAY	hsa05200: pathways in cancer	18	5.625	1.66*E* − 04	PTGER3, PTGS2, ERBB2, CDH1, GLI3, MMP2, CTNNB1, JUP, MAPK1, CBLC, ITGA6, RAC2, JUN, SLC2A1, RAC1, LAMC1, HHIP, FGF1
KEGG_PATHWAY	hsa04510: focal adhesion	14	4.375	2.41*E* − 05	ERBB2, TNC, ITGB5, FLNA, MYL9, CTNNB1, MAPK1, ITGA6, RAC2, JUN, RAC1, LAMC1, SPP1, PARVA
KEGG_PATHWAY	hsa05205: proteoglycans in cancer	13	4.0625	8.27*E* − 05	LUM, ERBB2, ITGB5, TLR4, MMP2, FLNA, CTNNB1, CBLC, MAPK1, SDC1, CD44, RAC1, MSN
KEGG_PATHWAY	hsa04151: PI3K-Akt signaling pathway	12	3.75	0.022876	MAPK1, SGK1, YWHAZ, ITGA6, TNC, RAC1, YWHAB, ITGB5, TLR4, LAMC1, FGF1, SPP1
KEGG_PATHWAY	hsa04810: regulation of actin cytoskeleton	11	3.4375	0.001989	MAPK1, ENAH, ITGA6, RAC2, CHRM3, RAC1, ITGB5, ITGB2, MSN, FGF1, MYL9
KEGG_PATHWAY	hsa05412: arrhythmogenic right ventricular cardiomyopathy (ARVC)	8	2.5	9.23E-05	JUP, ITGA6, DSG2, ITGB5, GJA1, DSP, CACNA2D3, CTNNB1

**Table 3:** The six clusters obtained from module analysis using MCODE.

Cluster	Score (density^∗^#nodes)	Nodes	Edges	Node IDs
1	6	9	24	CCL5, PPBP, APLNR, ANXA1, GPR65, F2RL1, PTGER3, CHRM3, APP
2	5	5	10	GBP6, HLA-DPA1, HLA-DPB1, CD44, IRF6
3	4.5	5	9	TOP2A, ENTPD3, RRM1, TYMS, RRM2
4	4	4	6	XYLT1, BGN, SDC1, CSPG4
5	3	3	3	LRRFIP1, FGF1, ERLIN2
6	3	3	3	ITGA6, YWHAB, YWHAZ

## Data Availability

The datasets used and/or analyzed during the current study are available from corresponding author.
